# Risk Factors of Cardiovascular and Cerebrovascular Events in Patients With Uraemia Complicated With Hypertension During Maintenance Haemodialysis Treatment

**DOI:** 10.7759/cureus.53411

**Published:** 2024-02-01

**Authors:** Awais Ahmed Nizami, Waqar Mustafa, Mamoon Qadir, Maria Shahzad, Hamid Iqbal, Anwar Ali, Sarosh Khan Jadoon, Amna Akbar, Sabahat Tasneem, Mohammad Saleem Khan

**Affiliations:** 1 Cath Lab, Shahida Islam Medical College, Lodhran, PAK; 2 Cardiology, Combined Military Hospital, Muzaffarabad, PAK; 3 Cardiology, Federal Government Polyclinic, Islamabad, PAK; 4 Cardiology, Kulsum International Hospital, Islamabad, PAK; 5 General Surgery, Combined Military Hospital, Muzaffarabad, PAK; 6 Emergency and Accident, District Headquarters Hospital, Muzaffarabad, PAK; 7 Public Health, Health Services Academy, Islamabad, PAK; 8 Medicine, District Headquar­ters Hospital, Kotli, PAK

**Keywords:** risk factors, cardiovascular and cerebrovascular events, maintenance haemodialysis, hypertension, uraemia

## Abstract

Introduction: This study aimed to investigate the risk factors associated with major adverse cardiovascular (group of events that affect heart and blood vessels) and cerebrovascular (events affecting blood vessels supplying the brain) events (MACCE) in patients with uraemia complicated with hypertension who required maintenance haemodialysis (MHD) treatment.

Methodology: Clinical data and laboratory indicators of 156 uraemia patients complicated with hypertension were collected and retrospectively analysed. The patients were admitted to a tertiary care hospital (Abbas Institute of Medical Sciences AIMS) in Muzaffarabad, Pakistan, from February 2018 to February 2022. The data was collected through consecutive sampling and patients were recruited after following the inclusion and exclusion criteria.

Results: Eighty-one out of 156 patients were not complicated with MACCE, and 75 patients were complicated with MACCE during the MHD treatment cycle, with an incidence of 48.08%. Compared to the non-MACCE group, the MACCE group’s diabetes, body mass growth rate, triglyceride (TG), NT-proBNP, standard deviation and coefficient of variance for systolic and diastolic blood pressure (SBP-SD, SBP-CV, DBP-SD, and DBP-CV) showed significant differences (P<0.05) between the groups. Diabetes, body mass growth rate, TG, NT-proBNP, SBP-SD, SBP-CV, DBP-SD, and DBP-CV with odds ratios of 3.074, 3.202, 2.188, 2.512, 2.357, 2.431, 2.299, and 2.062 respectively were risk factors for MACCE in uraemia patients with hypertension.

Conclusion: From the results of this study, we inferred that patients with uraemia and hypertension complicated by MACCE in the treatment cycle of MHD were related to diabetes, body mass growth rate, TG, NT-proBNP, SBP-SD, SBP-CV, DBP-SD, and DBP-CV.

## Introduction

Uraemia, short for the uremic stage of chronic kidney disease, is the terminal stage of the development of chronic renal failure and is also a common clinical syndrome in the advanced stages of various kidney diseases [1]. Haemodialysis can replace the human kidney to complete the body's metabolism, remove metabolic waste in the body, and maintain the balance of water, electrolytes, acids, and bases, ultimately ensuring the stability of the internal environment and achieving the purpose of prolonging the survival of patients. Haemodialysis is a widely used and effective method for the treatment of uraemia [2]. With the continuous development of blood purification technology, the survival rate of patients with uraemia has improved significantly [3]. However, in the process of maintenance haemodialysis (MHD), patients often develop hypertension (chronic rise in blood pressure). It increases the incidence of major adverse cardiovascular and cerebrovascular events (MACCE) and mortality [4]. Relevant research findings showed that cardiovascular disease and cerebrovascular diseases are important risk factors for death in MHD patients, with mortality risks of 36% and 11%, respectively [5]. In the treatment cycle of MHD, there are many factors that cause MACCE in uraemia patients with hypertension, but there has been no unified understanding thus far. Reducing the occurrence of MACCE in the treatment cycle of MHD is an urgent problem that needs to be solved. Analysis of the factors related to cardiovascular and cerebrovascular events in uraemia patients with hypertension is of great significance for guiding clinical preventive measures and reducing the occurrence of MACCE in the treatment cycle of MHD. Currently, there are few reports on MACCE in patients with uraemia and hypertension [6]. Therefore, this study retrospectively analysed the occurrence and influencing factors of MACCE in uraemia patients with hypertension treated with MHD. Data was obtained for the time period that ranged from February 2018 to February 2022 at a tertiary care hospital in Muzaffarabad, Pakistan. This study aimed to provide a more theoretical basis for the clinical treatment of uraemia and to guide clinical intervention measures to reduce the occurrence of MACCE. This can help improve the quality of life of MHD patients and reduce their mortality rates.

## Materials and methods

Clinical data and laboratory indicators of 156 patients admitted to a tertiary care hospital (Abbas Institute of Medical Sciences AIMS) in Muzaffarabad, Pakistan, from February 2018 to February 2022 were collected for retrospective analysis. Patients suffering from uraemia complicated with hypertension are patients with insufficient renal function and diagnosed with hypertension on the basis of World Health Organization (WHO) criteria [7-8].

Inclusion/exclusion criteria

Inclusion criteria were as follows: (1) confirmation of chronic kidney disease (CKD) stage 5 diagnostic criteria [7] and hypertension diagnostic criteria established by the World Health Organization (WHO) [8]; (2) indication for MHD and receiving MHD treatment; and (3) haemodialysis time > 12 months and haemodialysis performed three times a week for four hours each time. The exclusion criteria were as follows: (1) severe liver disease or chronic infectious wasting disease, (2) severe bleeding tendency, (3) mental disorders, (4) low treatment compliance, and (5) missing clinical information, blood biochemical indexes, blood lipid indexes, and blood pressure parameters. 

The timing and scheme of maintenance dialysis therapy: (1) Timing: K/DOQI recommended that when patients with an estimated glomerular filtration rate (eGFR) less than 15 ml/min/1.73 m2 or weekly urea Kt/V less than 2.0, at stage 5 of CKD, the nephrologist evaluated the benefits, risks, and disadvantages of initiating renal replacement therapy and began preparation for dialysis therapy. However, it is generally recommended that non-diabetic patients with eGFR less than 10 (mL/min/1.73 m2) should start dialysis, diabetic patients with eGFR less than 15 (ml/min/1.73 m2) should start dialysis, and some patients with renal failure with special comorbidities may need to start dialysis earlier [9]. (2) Dialysis regimen: haemodialysis was performed three times a week for 4 h each time. The blood flow rate was 200 - 250 ml/min and the dialysate flow rate of 500 ml/min. Vascular access included an autologous arteriovenous fistula or long-term jugular vein catheter.

Data collection

Patient medical records were reviewed, and clinical data and laboratory indicators, including sex, diabetes, hyperlipidaemias, hyperphosphatemia, smoking history, history of alcohol consumption, age, course of disease, BMI, percentage change in body mass during the study, dialysis age, plasma albumin, haemoglobin, blood calcium, serum inorganic phosphorus, serum sodium, calcium-phosphorus product, total cholesterol (TC), triglycerides (TG), high-density lipoprotein cholesterol (HDL-C), low-density lipoprotein cholesterol (LDL-C), serum creatinine, urea, uric acid, N-Terminal Pro-B-type natriuretic peptide (NT-proBNP), systolic blood pressure (SBP), systolic blood pressure-standard deviation (SBP-SD), systolic blood pressure-coefficient of variation (SBP-CV), diastolic blood pressure (DBP), diastolic blood pressure-standard deviation (DBP-SD), diastolic blood pressure-coefficient of variation (DBP-CV), and adiponectin, were recorded. SBP and DBP are measured at each patient visit and the values are noted on the patient history sheet. The values were obtained from the sheets and a mean of these values was used, standard deviation (SD) and coefficient of variation was then calculated.

Diagnostic criteria and grouping

Hypertension Diagnostic Criteria

Systolic blood pressure ≥ 140 mmHg and/or diastolic blood pressure ≥ 90 mmHg following repeated examinations should be referred to as hypertension according to the diagnostic criteria of the 2020 International Society of Hypertension Global Hypertension Practice Guidelines. Patients in whom SBP ≥ 140 mmHg and DBP ≥ 90 mmHg were persistent even after the use of antihypertensives were diagnosed with chronic hypertension [10].

Diagnostic Criteria for MACCE

Cardiovascular events were diagnosed according to the K/DOQI and clinical diagnosis and treatment guidelines, including myocardial infarction, unstable angina pectoris, coronary artery bypass grafting, heart failure requiring hospitalization, percutaneous coronary intervention, ischemic heart disease, malignant arrhythmia, and congestive heart failure (CHF). Cerebrovascular events included cerebral haemorrhage and stroke, cerebral ischemic stroke, cerebral infarction, and transient cerebral ischemia.

Grouping

During the treatment cycle, the number of patients with MACCE was counted. Patients with MACCE were included in the MACCE group, and those without MACCE were included in the non-MACCE group. The death toll was not included in the study.

Detection methods of relevant indicators

Blood Biochemical Indices and Serum Adiponectin

Six millilitres of fasting blood was extracted in the morning, and 0.3 mL of 3.84% citrate was added to 3 mL of the blood samples and centrifuged at 3000 revolutions per minute for 10 min. The plasma was then separated and stored at -30 degrees Celsius for examination. Blood lipid indices (total cholesterol, triglycerides, high-density lipoprotein cholesterol, and low-density lipoprotein cholesterol) were determined by enzyme colourimetry. Electrolytes (blood sodium, blood calcium, blood phosphorus), renal function indices (urea and blood creatinine), uric acid, and serum NT- proBNP were detected by conventional methods. Adiponectin levels were determined using radioimmunoassay.

Blood Pressure Variability (BPV)

**Figure 1:**
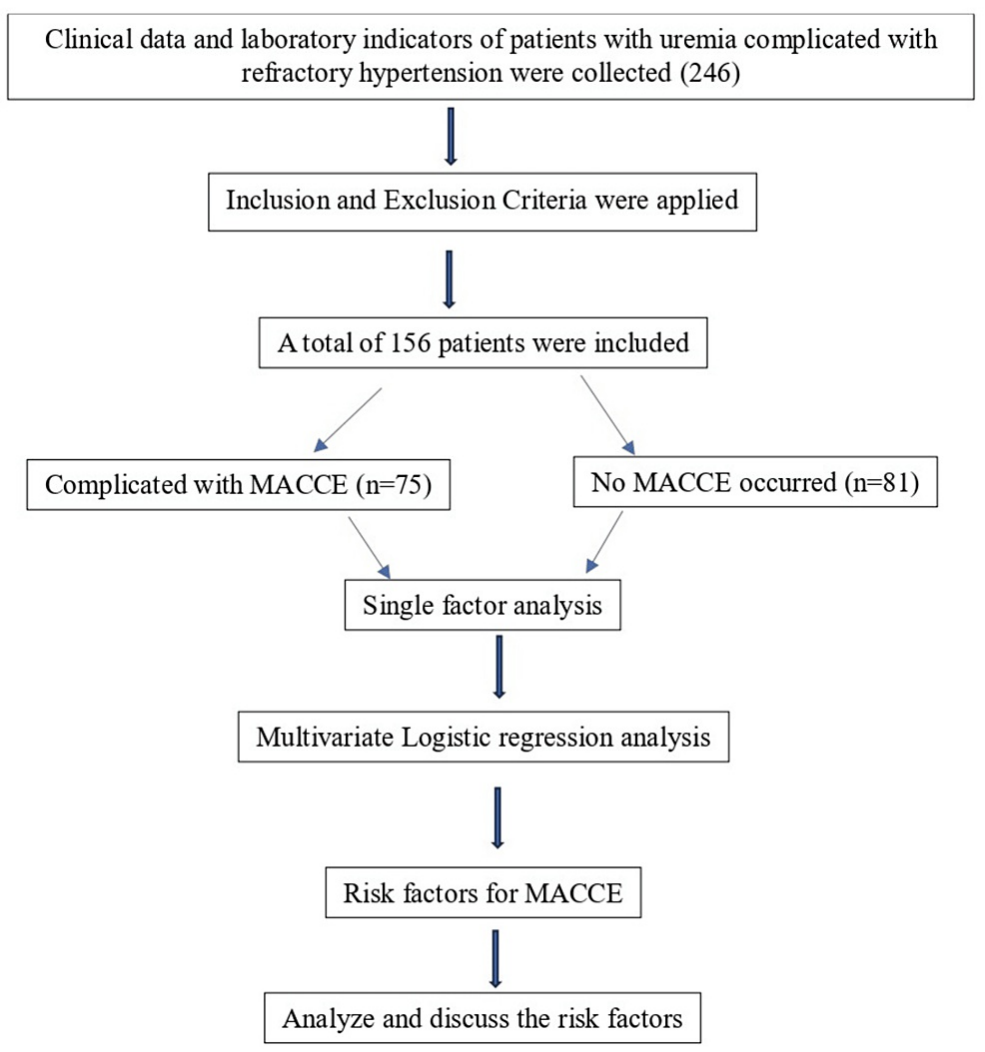
The technical roadmap of this study MACCE: Major adverse cardiovascular and cerebrovascular events

Ambulatory blood pressure monitoring (ABPM to measure SBP and DBP) was performed at each patient dialysis visit and the values were noted on a patient history sheet as a routine protocol of the hospital. According to the data, the blood pressure during the daytime was measured every 30 min from 09:00 to 22:00 at each dialysis visit. Referring to the research method of Rothwell et al. [11], the variability of systolic blood pressure (SBP) and diastolic blood pressure (DBP) was determined by the SD and coefficient of variability (CV). The standard deviation of the mean blood pressure values measured three times was denoted as SD, and CV= SD/mean blood pressure. According to the European Society of Cardiology (ESC) and the European Society of Hypertension (ESH: 24h mean blood pressure ≥ 130/80 mmHg, daytime mean blood pressure ≥ 135/85 mmHg, night mean blood pressure ≥ 120/70 mmHg, or night mean systolic blood pressure ≥ 125 mmHg. Dipper-type blood pressure was defined as nocturnal mean blood pressure ≥ 10% lower than diurnal mean blood pressure, whereas non-dipper-type blood pressure was defined as decreased blood pressure <10% [12]. The summary of the methodology is given in Figure 1.

Statistical Methods

IBM SPSS Statistics for Windows, Version 22 (Released 2013; IBM Corp., Armonk, New York, United States) was used for data analysis. Qualitative data were expressed as [n (%)], quantitative data were expressed as (x ± s), and t-tests were performed. Univariate and multivariate logistic regression models were applied to investigate the factors related to MACCE in patients with uraemia complicated by hypertension during the MHD treatment cycle, and P< 0.05 was statistically significant.

## Results

Following inclusion and exclusion criteria, a total of 156 patients were chosen from 246 patients for this study, of which 81 patients were not complicated with MACCE and 75 patients were complicated with MACCE during the MHD treatment cycle, with an incidence of 48.08% (Figure 1). Comparisons of sex, hyperlipidaemias, hyperphosphatemia, smoking history, drinking history, age, course of disease, BMI, dialysis age, plasma albumin, haemoglobin, blood calcium, blood phosphorus, blood sodium, calcium-phosphorus product, TC, LDL-C, HDL-C, urea, serum creatinine, uric acid, SBP, DBP, and adiponectin between the MACCE group and non-MACCE group (P> 0.05). Compared with the non-MACCE group, MACCE group’s diabetes, body mass growth rate, TG, NT-proBNP, SBP-SD, SBP-CV, DBP-SD, and DBP-CV showed significant differences (P< 0.05). The results of the statistical analysis are shown in Table 1.

**Table 1:** Univariate analysis of MACCE in uremic patients with hypertension SD: Standard deviation; CV: coefficient of variation; MACCE: major adverse cardiovascular and cerebrovascular events; NT-proBNP: N-terminal Pro-B-type natriuretic peptide; SBP-SD: systolic blood pressure-standard deviation; SBP-CV: systolic blood pressure-coefficient of variation; DPP-SD:  diastolic blood pressure-standard deviation; DBP-CV: diastolic blood pressure-coefficient of variation

Influence factor	MACCE group(n=75)	non-MACCE group(n=81)	t/c2	P
Gender (Female/male)	28/47	29/52	0.039	0.843
Concomitant disease				
Diabetes	24(32.00)	4(4.94)	19.365	＜0.001
Hyperlipidemia	18(24.00)	23(28.40)	0.388	0.533
Hyperphosphatemia	8(10.67)	7(8.64)	0.184	0.668
Smoking history	25(33.33)	29(35.80)	0.105	0.746
Age(year)	54.22±8.39	53.87±7.16	0.281	0.779
Course of disease(year)	2.58±0.57	2.61±0.42	0.376	0.707
BMI (kg/m^2^)	22.53±2.04	22.49±2.13	0.120	0.905
Growth rate of body mass (%)	7.02±1.33	4.86±1.25	10.430	<0.001
Dialysis age(month)	8.43±2.34	8.52±2.16	0.250	0.803
Blood biochemical indexes				
Plasma-albumin (g/L)	38.22±3.39	38.41±3.10	0.366	0.715
Hemoglobin (g/L)	113.25±12.48	112.59±13.32	0.319	0.750
Blood calcium (mmol/L)	2.28±0.22	2.31±0.17	0.957	0.340
Serum inorganic phosphorus (mmol/L)	2.48±0.52	2.42±0.43	0.788	0.432
Serum sodium (mmol/L)	137.28±4.19	136.85±4.25	0.636	0.526
Calcium*phosphorus (mmol^2^ /L^2^)	5.48±0.62	5.39±0.47	1.026	0.306
TG (mmol/L)	1.29±0.42	1.13±0.30	2.753	0.007
TC (mmol/L)	4.42±0.58	4.38±0.29	0.551	0.583
LDL-C(mmol/L)	2.74±0.92	2.66±1.03	0.510	0.611
HDL-C(mmol/L)	1.01±0.11	0.99±0.08	1.306	0.194
Urea(mmol/L)	34.25±6.38	33.74±7.41	0.459	0.647
Serum creatinine(μmol/L)	915.26±25.18	917.02±20.27	0.483	0.630
Uric Acid(μmol/L)	402.55±33.16	405.19±28.87	0.531	0.596
NT-proBNP(ng/L)	6.28±0.47	5.88±0.62	4.514	<0.001
Blood-pressure parameter acquisition				
SBP	143.45±12.28	142.89±11.47	0.295	0.769
DBP	96.21±8.84	95.52±9.13	0.479	0.633
SBP-SD	19.33±4.26	9.25±3.02	17.150	<0.001
SBP-CV	13.17±3.21	5.87±1.85	17.560	<0.001
DBP-SD	10.82±3.29	5.44±1.62	13.100	<0.001
DBP-CV	14.16±4.23	6.24±2.05	15.050	<0.001
Adiponectin	5.37±1.64	5.42±1.38	0.2066	0.837

**Figure 2:**
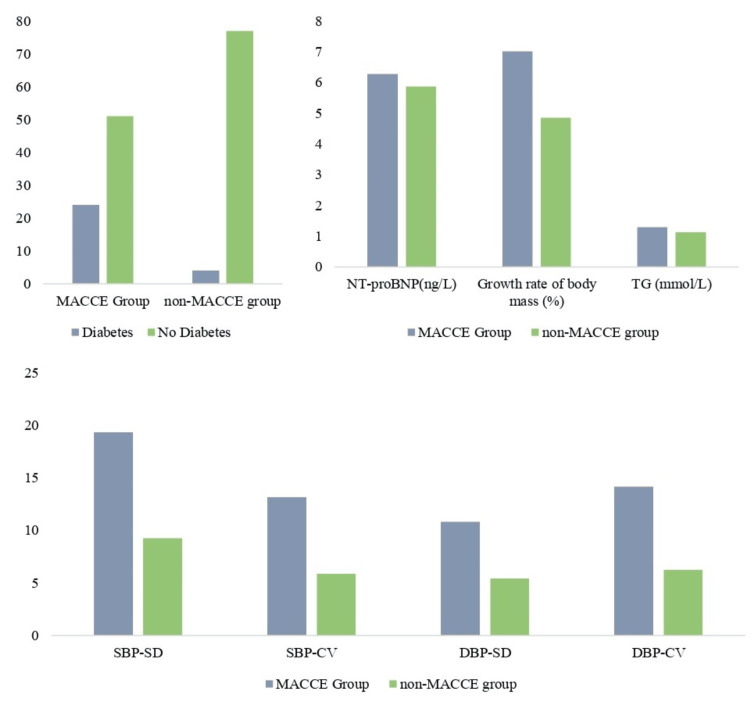
Diabetes distribution and the levels of body weight growth rate, TG, NT-proBNP, SBP-SD, SBP-CV, DPP-SD, and DBP-CV in the two groups MACCE: Major adverse cardiovascular and cerebrovascular events; TG: triglycerides; NT-proBNP: N-terminal Pro-B-type natriuretic peptide; SBP-SD: systolic blood pressure-standard deviation; SBP-CV: systolic blood pressure-coefficient of variation; DPP-SD:  diastolic blood pressure-standard deviation; DBP-CV: diastolic blood pressure-coefficient of variation

Diabetes distribution and body weight growth rate, TG, NT-proBNP, SBP-SD, SBP-CV, DPP-SD, and DBP-CV in the two groups are shown in Figure 2.

**Figure 3:**
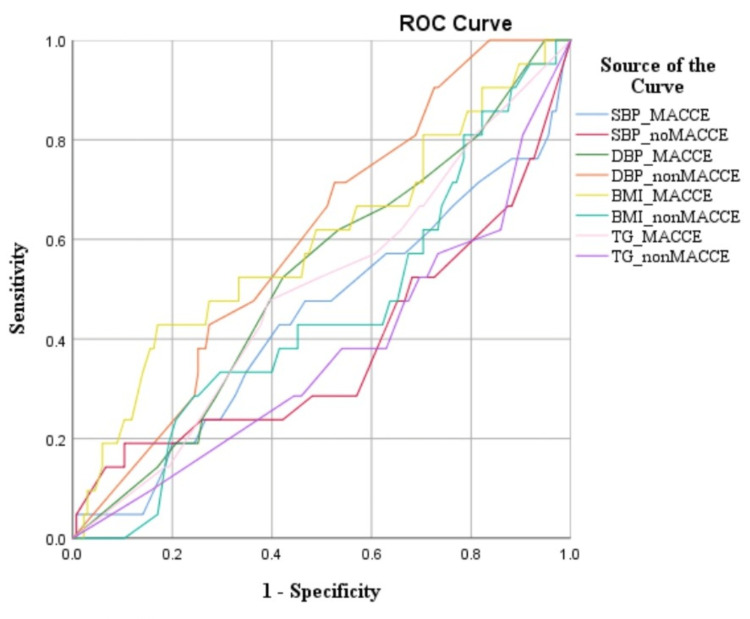
Receivers' operating curve MACCE: Major adverse cardiovascular and cerebrovascular events

The items with statistical significance in single-factor analysis (diabetes, body mass growth rate, TG, NT-proBNP, SBP-SD, SBP-CV, DPP-SD, and DBP-CV) were taken as independent variables, and the ROC curve was used to find the optimal truncation value of continuous variables in the independent variables. The ROC curve analysis results are shown in Figure 3.

The optimal truncation value and assignment of the independent variables are shown in Table 2. 

**Table 2:** Risk factors and evaluation of MACCE in uraemia patients with hypertension MACCE: Major adverse cardiovascular and cerebrovascular events; TG: triglycerides; NT-proBNP: N-terminal Pro-B-type natriuretic peptide; SBP-SD: systolic blood pressure-standard deviation; SBP-CV: systolic blood pressure-coefficient of variation; DPP-SD:  diastolic blood pressure-standard deviation; DBP-CV: diastolic blood pressure-coefficient of variation

Factor	Code	Assignment
Diabetes	X1	0=Have，1=Not have
Growth rate of body mass	X2	0=<5.54%，1=≥5.54%
TG	X3	0=<1.40 mmol/L，1=≥1.40 mmol/L
NT-proBNP	X4	0=<5.82 ng/L,1=≥5.82 ng/L
SBP-SD	X5	0=<13.52，1=≥13.52
SBP-CV	X6	0=<8.63，1=≥8.63
DBP-SD	X7	0=<8.14, 1=≥8.14
DBP-CV	X8	0=<8.82, 1=≥8.82

Using multivariate logistic regression analysis, diabetes, body mass growth rate ≥5.54%, TG ≥1.40 mmol/L, NT-proBNP ≥5.82 ng/L, SBP-SD ≥13.52, SBP-CV ≥ 8.63, DBP- SD ≥8.14, and DBP-CV ≥8.82) were risk factors for MACCE in uraemia patients with hypertension, and the differences were statistically significant (p<0.05) as shown in Table 3.

**Table 3:** Multivariate logistic regression analysis results TG: Triglycerides; NT-proBNP: N-terminal Pro-B-type natriuretic peptide; SBP-SD: systolic blood pressure-standard deviation; SBP-CV: systolic blood pressure-coefficient of variation; DPP-SD:  diastolic blood pressure-standard deviation; DBP-CV: diastolic blood pressure-coefficient of variation

Independent variable	β	Waldχ^2^	P	OR(95%CI)
Diabetes	3.074	12.458	<0.001	21.633(3.924–119.263)
Growth rate of body mass	3.202	21.268	<0.001	24.578(6.303–95.835)
TG	2.188	7.428	0.006	8.917(1.849–43.007)
NT-proBNP	2.512	13.148	<0.001	12.329(3.171–47.926)
SBP-SD	2.357	13.149	<0.001	10.560(2.954–37.756)
SBP-CV	2.431	12.233	<0.001	11.370(2.912–44.397)
DBP-SD	2.299	11.138	0.001	9.967(2.583–38.460)
DBP-CV	2.062	10.202	0.001	7.860(2.218–27.852)

## Discussion

Hypertension in haemodialysis patients is related to renal failure and damage and it is important to investigate essential hypertension and renal hypertension caused by kidney disease as well as factors related to haemodialysis [13]. Blood pressure during dialysis treatment was related to the risk of death; high or low systolic blood pressure after dialysis was associated with an increased risk of cardiovascular death, and mortality was significantly increased when the diastolic blood pressure was greater than 109 mmHg [14]. Currently, MACCE is one of the main causes of death in uraemia patients during MHD treatment [15]. In the present study, patients with uraemia complicated by hypertension were included, and the incidence of MACCE was found to be 48.08% indicating higher blood pressure in the group.

The results of this study showed that diabetes body mass growth rate ≥ 5.54%, TG ≥ 1.40 mmol/ L, NT-proBNP ≥ 5.82 ng/L, SBP-SD ≥ 13.52, SBP-CV ≥ 8.63, DBP-SD ≥8.14, and DBP-CV ≥ 8.82 were risk factors for MACCE in uraemia patients with hypertension. Patients with diabetes are more prone to developing MACCE [16]. Hypertension has been proven to be a cardiovascular risk factor [17]. Other studies have shown that diabetes increases the risk of vascular diseases [13]. Under the double impact of hypertension and diabetes, the risk of MACCE in patients with uraemia has greatly increased. Hypertension is related to body mass index and cardiovascular and cerebrovascular diseases [18]. Hypertension promotes an increase in the body mass of patients, and the incidence of MACCE is related to an increase in body mass index, which could increase the incidence of hypertension, hyperlipidaemias, arteriosclerosis, and diabetes, thus causing the formation of MACCE [19]. This indicates that weight gain increases the risk of MACCE. The results of this study showed that the risk of MACCE was significantly increased when the growth rate of body mass exceeded 5.54%. Lipid and complex carbohydrate accumulation, haemorrhage, thrombosis, fibrous hyperplasia, and calcareous deposition are the basis of atherosclerosis [20]. TG is an important component of lipid substances, and increased TG levels indicate increased lipid accumulation, promoting the formation and development of atherosclerosis and leading to an increase in the incidence of MACCE. NT-proBNP is a serum marker that is synthesized mainly by cardiomyocytes. When the ventricular wall tension is too high, it accelerates the synthesis and secretion of NT-proBNP by cardiomyocytes. The discharge capacity of NT-proBNP in patients with uraemia decreases, so the concentration of NT-proBNP in the plasma increases. Decreased renal function in haemodialysis patients significantly increases the risk of cardiovascular events [21].

BPV is a clinical indicator reflecting the degree of blood pressure fluctuation over a certain period. The clinical blood pressure SD and coefficient of variation (CV) are commonly used to reflect the size of the BPV. Recent studies have found that BPV is significantly positively correlated with the occurrence of MACCE [22-23]. The results of this study showed that SBP-SD, SBP-CV, DBP-SD, and DBP-CV were risk factors for MACCE in patients with uraemia complicated by hypertension during the treatment cycle of MHD, which was consistent with relevant research results [24]. BPV is an important risk factor for target organ damage in patients with hypertension, and SBP-SD and DBP-SD are the main indices reflecting BPV. The instability of SBP-SD and DBP-SD increases the risk of cardiovascular adverse events [25,26]. Insufficient data exist explaining the correlation between blood pressure levels and prognosis in dialysis patients. Blood pressure regulation is imperative both in pre-and post-procedures but also during dialysis [27]. Therefore, we guessed that timely and effective intervention measures for high-risk individuals could reduce the incidence of MACCE so that we could ensure the safety of dialysis and reach a better prognosis.

Limitations

This was a single-centre, retrospective study with a limited number of cases. This may have led to a bias in the statistical results and there is no external validation for the study. Therefore, a multicentre study with many samples should be conducted to improve the reliability of the research conclusions.

## Conclusions

Patients with uraemia and hypertension complicated with MACCE in the treatment cycle of MHD were related to diabetes, body mass growth rate, TG, TC, NT pro-BNP, SBP-SD, SBP-CV, DBP-SD, and DBP-CV. Therefore, early screening of high-risk patients and positive intervention measures should be performed to reduce the risk of MACCE and ensure dialysis safety. Blood pressure variability can be used as a clinical indicator to monitor fluctuation in BP. The treatment regimens should focus on maintaining systolic and diastolic blood pressure keeping the risk factors in check.

## Appendices

**Table 4:** Data Set

age-1	age-2	sex-1	sex-2	Diabetes-1	Diabetes-2	Hyperlipidemia-1	Hyperlipidemia-2	Hyperphosphatemia-1	Hyperphosphatemia-2	Smoking history-1	Smoking history-2	TIME-1 year	TIME-2 year	SBP-1	SBP-2	DBP-1	DBP-2	BMI_1	BMI_2	DIALYSIS MONTHS-1	DIALYSIS MONTHS-2	NT-proBNP-1	NT-proBNP-2	Hemoglobin(g/L) PG	Hemoglobin(g/L) CG	plasma Albumin-1	plasma Albumin-2	SerumNa-1	SerumNa-2	TC(mmol/L)-1	TC(mmol/L)-2	TG(mmol/L)-1	TG(mmol/L)-2	HDL-C(mmol)-1	HDL-C(mmol/L)-2	LDL-C(mmol/L)-1	LDL-C(mmol/L)-2	Blood calcium(mmol/L) PG	Blood calcium(mmol/L) CG	Serum potassium(mmol/L)-1	Serum potassium(mmol/L)-2	phosphorus(mmol/L)-1	phosphorus(mmol/L)-2	UA(μmol/L)-1	UA(μmol/L)-2	urea-1	Urea-2	creatinine-1	creatinine-2
42	49	M	F	0	1	1	1	0	1	1	0	2.4	1.3	136	140	92	60	24.69	22.92	13	14	4500	1615	125.6	125.4	40.82	39.53	114.946	100.25	4.88	4.99	2.06	1.5	1.51	1.8	2.377	2.897	2.21	2.37	4.35	3.61	5.2	4.59	232.02	209.27	34.59	33.92	547.06	1012.33
38	52	M	M	1	0	1	0	0	0	0	1	1.7	1.4	127	132	95	65	31.65	22.76	14	13	5250	1200	113.6	116.3	39.52	40.97	150.314	106.104	5.16	4.15	1.88	1.8	1.7	1.92	2.027	2.597	2.36	2.28	3.91	3.39	4.66	4.89	212.27	203.97	34.42	36.99	1165.56	1116.50
48	59	M	M	1	1	1	1	0	0	1	1	2.6	1.2	133	138	77	62	23.83	23.2	13	14	4635	1740	125.4	118.2	42.38	42.53	150.314	123.788	4.16	3.94	0.93	1.62	1.8	1.56	2.757	2.247	2.22	2.22	4.24	3.21	4.8	4.59	182.07	195.94	35.56	34.7	583.33	1217.46
69	51	M	M	1	1	1	1	1	1	1	1	2.4	1.2	128	133	70	70	27.98	21.48	13	14	4920	2000	119.6	125.4	42.21	42.77	141.472	106.104	5.02	4.54	2.06	0.67	1.52	1.75	1.787	2.007	2.23	2.46	3.96	3.26	4.91	4.84	168.87	150.87	34.15	33.65	1364.24	561.86
63	57	M	F	1	1	1	1	0	1	1	1	2.4	1.2	130	135	71	79	25.69	21.32	13	14	4605	1625	121.6	129.4	42.58	39.67	114.946	97.262	4.08	4.85	2.06	1.24	1.7	1.85	2.027	2.977	2.46	2.18	3.91	3.21	4.66	4.73	221.97	194.27	33.7	34.25	1236.84	849.27
46	69	F	F	0	1	1	1	1	0	0	0	1.9	1.3	129	134	78	80	24.26	26.83	14	13	3315	1650	113.6	118.2	42.38	39.53	106.104	100.25	4.11	4.54	0.93	1.62	1.8	2.06	2.377	2.247	2.22	2.22	4.12	3.53	5.2	4.86	191.77	203.97	35.2	34.09	550.80	965.41
43	63	M	M	0	0	0	0	1	0	0	1	2.1	1.2	135	139	80	66	22.01	21.48	14	13	5445	1830	132.6	113.6	40.81	42.36	107.56	79.578	4.71	4.85	2.06	1.01	1.85	1.56	2.677	2.927	2.18	2.36	3.96	3.26	4.8	4.92	168.87	164.07	34.59	33.92	1044.44	826.44
44	60	M	M	1	0	1	1	1	1	1	1	1.8	1.3	135	138	90	69	29.11	22.01	14	13	4605	1650	125.4	125.4	42.21	42.77	106.104	123.788	5.02	4.54	1.05	0.79	1.51	1.8	2.027	2.597	2.22	2.34	3.91	3.26	4.93	4.59	168.87	194.27	37.49	35.06	810.81	617.89
42	52	F	M	1	1	1	1	1	0	0	1	2.4	1.3	126	131	95	60	24.11	22.4	15	13	6135	1840	118.2	119.6	42.38	42.73	108.25	79.578	3.99	4.99	0.93	1.62	1.7	1.85	1.787	2.247	2.21	2.22	4.09	3.54	4.8	5.13	221.97	206.05	34.15	33.65	1044.44	1054.68
56	59	M	M	0	0	1	0	0	0	1	1	1.8	1.2	135	139	95	80	32.8	22.92	12	12	4830	1600	125.4	121.6	40.82	39.75	114.946	100.25	5.02	4.54	1.76	1.8	1.51	1.73	2.707	2.977	2.18	2.21	3.91	3.26	4.66	4.84	168.87	195.94	34.42	33.2	1035.29	1219.46
44	87	M	M	0	1	1	1	0	0	1	1	1.7	1.2	128	133	72	66	29.58	24.8	13	14	3900	1615	116.3	113.6	39.6	40.97	109.52	97.262	5.16	3.94	1.05	1.5	1.8	1.56	2.027	3.047	2.36	2.36	4.16	3.42	4.96	4.86	212.27	164.07	35.2	34.7	1236.84	1140.54
63	42	F	F	1	1	1	1	1	1	0	0	2.0	1.3	125	130	95	69	34.4	22.36	13	14	5775	2000	119.6	125.4	42.58	42.53	106.104	88.42	5.16	4.39	1.78	0.79	1.68	1.56	2.707	2.717	2.46	2.46	3.91	3.21	3.16	3.09	168.87	203.97	36.59	34.09	1113.51	965.41
46	71	M	M	1	1	1	1	1	0	1	1	1.8	1.2	129	135	95	60	25.3	22.76	14	12	4890	1640	114.9	116.3	39.38	42.36	159.156	114.946	4.88	4.71	1.5	0.67	1.85	1.75	2.027	2.007	2.28	2.18	3.96	3.46	4.66	4.59	168.87	194.27	37.24	36.99	1085.53	561.86
46	54	M	M	1	1	1	1	1	0	1	1	2.4	1.3	135	140	77	80	27.73	22.66	12	12	4590	2010	116.3	118.2	42.21	42.53	132.63	114.946	4.08	4.99	0.93	0.77	1.7	1.85	1.787	2.597	2.22	2.22	4.24	3.21	5.2	4.59	212.27	173.77	34.15	33.65	516.67	657.26
32	59	F	M	0	0	0	0	1	1	0	1	1.8	1.2	127	132	76	70	24.81	24.71	12	12	4590	1915	125.4	125.4	40.82	41.73	95.45	61.894	5.02	3.91	2.06	1.39	1.8	1.61	2.377	2.247	2.18	2.21	3.96	3.26	5.14	4.82	191.77	150.87	35.2	33.92	970.59	1210.85
56	56	F	M	0	0	1	0	1	1	1	1	2.1	1.2	135	139	78	73	20.59	25.2	11	12	4480	1530	125.6	129.4	42.62	40.96	132.63	97.262	4.11	3.82	1.88	1.62	1.87	1.85	2.027	2.977	2.36	2.33	3.91	3.21	4.8	4.73	182.07	164.07	34.42	34.7	695.36	826.44
51	59	F	M	0	0	0	0	0	0	0	1	1.4	1.2	130	135	80	70	24.89	24.54	14	12	4298	2000	127.6	118.2	40.81	42.53	114.946	100.25	4.56	3.91	1.65	1.62	2.01	1.92	1.787	2.007	2.46	2.22	4.31	3.42	4.66	5.13	221.97	203.97	37.49	36.09	512.44	1151.97
76	45	M	M	1	0	1	1	1	0	1	0	2.6	1.2	122	127	72	80	28.06	26	11	12	5446	1825	125.4	119.6	42.38	40.97	106.104	97.262	3.99	4.99	1.03	0.79	1.51	1.73	2.757	2.977	2.33	2.18	3.91	3.21	4.93	3.09	224.05	150.87	33.7	36.74	1092.72	1022.75
44	77	M	M	1	1	1	1	0	1	1	1	1.8	1.2	130	135	73	69	31.25	22.13	11	12	4872	1635	116.3	114.9	40.82	42.73	79.578	123.788	5.16	4.54	1.05	1.39	1.8	1.9	2.027	2.007	2.18	2.37	4.12	3.61	4.93	4.86	212.27	194.27	34.15	33.65	1101.60	682.18
73	57	M	F	1	1	1	1	0	0	1	0	1.7	1.3	129	133	95	80	25.78	22	13	14	4550	1800	119.6	127.6	41.58	39.75	114.946	132.63	5.16	4.99	2.06	1.8	1.75	1.75	1.787	2.597	2.34	2.22	4.19	3.21	4.66	5.07	168.87	203.17	37.22	34.7	679.14	1064.35
86	43	M	F	1	0	1	1	1	0	1	1	1.8	1.2	133	138	94	60	25.65	23.58	13	14	4242	1805	132.6	132.6	42.38	42.53	150.314	100.25	4.11	4.52	0.93	1.62	1.56	1.85	2.377	2.247	2.46	2.08	4.24	3.26	3.16	4.86	232.02	164.07	34.59	33.92	572.22	1217.46
51	72	M	M	1	1	0	1	0	1	1	1	2.4	1.3	130	135	79	65	22.83	26.83	14	12	5082	1910	125.4	125.4	39.6	40.97	108.45	97.262	4.71	4.85	1.88	0.98	1.51	1.92	2.027	2.897	2.37	2.22	3.96	3.54	5.21	4.59	182.07	214.02	33.7	36.99	970.59	1036.14
74	77	F	F	1	0	1	0	0	1	1	0	2.0	1.2	127	132	77	62	23.75	22.53	14	12	4312	1455	118.2	118.2	42.21	42.73	114.946	114.946	4.16	4.54	1.76	0.67	1.7	2.06	1.787	2.597	2.22	2.28	4.23	3.21	4.8	5.13	221.97	203.97	37.21	33.2	1320.51	1126.37
66	51	M	F	1	1	1	1	1	0	1	0	1.8	1.3	128	133	74	70	23.32	26.4	11	12	5124	1830	116.3	125.4	42.38	42.36	79.578	77.8096	4.88	4.39	1.27	1.8	1.52	1.56	2.677	2.247	2.18	2.46	3.91	3.46	4.91	5.14	182.07	194.27	34.15	33.65	561.50	1054.68
68	68	F	M	1	1	1	1	0	0	0	1	2.6	1.2	135	140	95	80	27.85	21.74	11	12	4844	1640	137.8	116.3	42.58	42.53	114.946	100.25	4.71	3.91	1.27	1.8	1.8	1.85	2.027	2.007	2.18	2.22	3.96	3.42	4.66	4.59	221.17	164.07	34.42	36.72	1113.51	973.87
68	72	M	M	1	1	1	1	0	1	1	1	1.4	2.4	127	132	74	60	23.83	23.44	11	12	4312	1555	125.6	119.6	40.81	42.77	150.314	115.49	4.08	4.99	2.06	0.67	1.51	1.8	2.827	2.247	2.22	2.36	4.31	3.21	4.93	3.09	212.27	150.87	33.7	34.09	1105.88	719.37
41	61	M	F	1	1	0	1	0	1	1	1	1.6	1.9	125	129	95	65	27.07	25.6	11	12	4500	1900	119.6	114.9	42.38	42.36	132.63	70.736	3.99	4.54	1.65	1.8	2.01	1.61	1.787	2.007	2.36	2.18	4.12	3.26	5.2	4.86	168.87	173.77	34.75	33.2	516.67	965.41
48	59	M	M	0	0	0	0	0	1	1	1	1.8	2.1	126	130	75	62	34.58	25.2	14	12	5250	1540	132.6	116.3	40.82	40.97	106.104	123.788	4.71	3.91	1.88	0.79	1.87	1.56	2.027	2.597	2.46	2.22	4.24	3.21	4.66	3.09	212.27	194.27	37.24	36.71	935.32	590.46
56	49	M	F	1	1	1	0	1	1	1	0	2.4	1.4	128	132	95	80	27.27	22.76	11	12	4635	1750	124.8	125.4	39.52	42.36	123.788	106.104	4.11	3.82	0.93	0.67	1.68	1.75	2.757	2.247	2.33	2.34	3.91	3.21	4.89	4.73	182.47	203.97	34.15	33.65	1165.56	1332.85
40	77	M	F	1	1	1	1	1	0	0	1	2.5	1.8	128	133	76	69	23.33	25.1	14	12	4920	1730	116.3	125.6	42.38	39.67	132.63	97.262	4.56	4.85	1.05	1.5	1.8	1.57	2.377	2.977	2.22	2.23	4.09	3.54	3.16	4.59	212.27	194.27	34.75	33.92	1364.24	1104.71
39	69	F	M	1	0	0	0	1	0	0	0	2.7	1.7	135	140	78	65	24	26.09	14	12	4605	1615	118.2	127.6	39.38	42.73	150.314	79.578	4.71	3.99	1.76	0.79	1.85	1.85	2.027	2.007	2.18	2.22	3.91	3.54	5.14	5.13	182.07	150.87	35.56	33.2	802.14	566.28
54	58	M	M	1	1	0	1	1	0	1	1	1.7	1.8	128	132	78	80	23.05	24.9	14	12	3315	1550	132.6	125.4	42.21	39.75	123.788	100.25	5.16	4.99	2.06	1.8	1.87	1.56	1.787	2.247	2.08	2.18	4.17	3.56	4.66	4.84	221.97	194.27	34.42	34.25	547.06	989.81
56	72	F	F	1	1	1	1	1	1	0	0	1.8	2.4	133	138	79	66	27.73	23.58	11	12	5445	1830	125.4	116.3	42.38	40.96	141.472	123.788	5.16	4.39	1.5	1.62	1.52	2.06	2.827	2.597	2.46	2.36	4.24	3.42	4.8	4.74	182.07	164.07	36.59	36.74	567.57	1217.46
40	62	F	F	1	1	1	1	0	1	0	1	2.5	2.4	130	136	80	69	24.58	26	13	14	4605	2025	116.3	119.6	40.81	42.53	106.104	115.49	4.71	3.91	1.88	0.77	1.52	1.92	2.027	2.897	2.22	2.37	4.12	3.21	4.93	4.86	182.07	194.27	34.15	33.65	627.78	951.22
51	87	M	M	1	1	1	1	0	0	1	1	2.6	2.1	127	132	80	60	26.17	25.78	14	12	6135	1600	113.6	132.6	42.58	41.73	106.104	123.788	3.99	3.94	1.05	1.62	1.7	1.73	1.787	2.247	2.18	2.46	3.91	3.42	3.16	3.09	212.27	194.27	37.22	34.25	550.80	580.04
44	57	F	F	0	0	0	0	0	0	0	0	2.2	1.2	136	141	75	79	29.54	23.58	13	12	4830	1740	125.6	125.4	42.38	42.53	114.946	100.25	4.88	4.2	0.93	1.24	1.8	1.85	2.677	2.007	2.36	2.22	3.96	3.21	4.91	4.59	227.27	150.87	34.59	35.06	1355.26	965.41
38	77	M	M	1	0	0	0	1	1	1	1	2.4	1.3	135	139	77	66	26.88	24	13	13	3900	1600	127.6	118.2	39.6	40.97	97.262	106.104	4.71	4.85	1.13	1.62	1.52	1.9	2.027	2.927	2.28	2.08	3.91	3.54	4.8	5.13	191.77	203.17	36.39	33.92	1035.29	1207.57
44	51	M	M	0	0	0	0	1	0	1	1	2.4	2.1	130	135	95	69	28.8	25.3	13	13	5775	1845	132.6	116.3	40.82	42.53	132.63	88.42	4.69	4.99	2.06	1.5	1.52	1.92	2.377	2.247	2.22	2.33	4.16	3.26	4.66	5.07	168.87	194.27	34.42	36.09	561.50	710.58
76	85	M	F	1	1	1	0	1	1	0	1	1.6	2.6	130	135	76	60	30.45	21.88	11	12	4890	1820	118.2	137.8	42.38	39.75	150.314	115.49	4.08	3.91	1.65	1.62	1.75	1.57	1.787	2.597	2.23	2.22	4.24	3.46	5.2	4.59	221.17	164.07	33.7	33.65	833.33	1054.68
48	80	F	F	1	1	1	1	0	1	0	0	2.0	2.6	132	139	95	80	27.1	22.39	13	13	4590	1545	121.9	125.6	41.54	42.53	97.262	100.25	4.71	3.91	1.88	1.8	1.87	1.59	2.027	2.007	2.46	2.18	4.35	3.21	4.93	4.73	182.07	209.27	37.21	36.72	625.00	566.28
58	71	F	M	1	1	1	1	0	1	0	1	6.1	4.3	127	132	75	66	25.65	20.45	13	13	4590	1700	125.4	119.6	40.81	42.73	106.104	97.262	3.99	4.99	1.76	1.39	1.51	1.75	2.757	2.247	2.18	2.23	4.31	3.42	5.21	4.92	221.97	203.97	35.2	34.09	941.18	594.79
70	42	M	M	1	1	1	0	1	0	1	0	4.8	5.3	126	130	95	69	26.4	23.98	11	12	4256	1625	124.6	132.6	42.21	42.53	114.946	106.104	5.02	4.39	1.5	0.67	1.7	1.85	2.707	2.977	2.22	2.34	4.24	3.54	3.16	3.09	168.87	214.02	35.56	35.89	695.36	1217.46
61	77	M	M	0	1	0	1	0	1	1	1	6.0	5.5	130	135	75	60	23.63	25.6	13	13	4242	1600	125.6	124.8	42.62	42.77	159.156	123.788	5.16	4.54	0.93	1.39	1.8	1.57	2.027	2.927	2.46	2.22	4.12	3.21	4.66	4.84	182.07	194.27	36.59	33.92	1245.03	895.76
61	64	F	F	1	1	1	1	0	0	0	0	5.6	4.4	126	131	76	66	24.69	26.4	11	12	3416	2000	131.2	116.3	40.82	42.53	141.472	132.63	5.16	4.99	1.88	0.79	2.01	1.52	1.787	2.247	2.22	2.36	3.91	3.61	4.99	5.13	212.27	164.07	34.15	33.2	970.59	1207.57
39	77	M	M	1	1	1	1	1	1	1	0	3.7	6.8	125	129	77	70	26.48	22.3	13	13	5376	1810	137.8	118.2	42.58	39.75	106.104	97.262	4.56	4.85	2.06	0.77	1.51	1.43	2.377	2.007	2.36	2.46	4.19	3.46	4.91	4.59	224.05	194.27	34.42	36.71	1236.84	1031.63
42	57	M	M	1	1	1	1	1	0	1	1	5.9	21.1	127	131	75	80	29.11	25.61	13	13	4494	1930	118.2	132.6	42.21	42.53	96.3778	79.578	4.88	3.82	1.88	1.39	1.75	1.56	2.027	2.897	2.22	2.28	4.23	3.42	4.66	4.86	168.87	173.77	35.2	34.7	615.89	736.69
48	63	M	F	0	0	0	0	1	1	1	1	26.8	14.5	156	160	76	60	26.75	20.82	11	12	3360	1250	119.6	125.4	39.6	40.96	141.472	106.104	4.11	4.54	1.05	1.8	1.8	1.75	1.787	2.247	2.37	2.22	4.24	3.21	4.93	5.13	168.87	150.87	37.22	35.06	1044.44	930.35
58	77	F	M	1	1	1	1	1	0	0	1	14.0	4.8	135	140	95	65	22.4	25.2	14	13	2912	1800	121.9	116.3	40.81	42.53	132.63	97.262	5.02	3.99	1.76	1.01	1.68	1.85	2.497	2.597	2.33	2.18	4.16	3.21	4.8	4.73	191.77	194.27	34.59	36.09	550.80	527.96
61	62	M	F	1	1	1	1	0	1	1	1	5.6	6.7	126	131	94	62	25.37	26	11	12	3472	1500	125.4	113.6	41.54	42.53	97.262	123.788	3.99	3.91	1.88	0.77	1.51	1.57	2.027	2.007	2.22	2.37	4.24	3.26	5.2	5.14	182.07	194.27	34.15	33.65	1245.03	822.31
46	70	F	F	0	1	0	1	0	1	0	0	8.5	9.5	132	138	75	70	29.69	22.27	14	13	4564	1815	124.6	125.6	42.38	41.69	114.946	100.25	4.16	4.99	1.65	1.39	2.01	1.75	2.757	2.247	2.18	2.22	3.91	3.65	4.66	4.59	212.27	164.07	35.2	33.92	725.71	1052.79
56	44	M	M	0	0	1	0	1	0	1	0	5.1	6.9	126	131	93	80	23.63	26.4	14	13	4074	2000	125.6	127.6	39.38	39.75	150.314	106.104	4.08	4.54	0.93	1.8	1.7	1.85	1.787	2.977	2.34	2.21	4.12	3.26	5.21	3.09	221.97	209.27	34.42	34.7	1105.88	1104.71
48	53	F	M	1	1	1	0	0	1	0	1	2.6	2.5	152	112	80	60	27.27	24.9	15	13	4650	2000	132.6	132.6	42.21	40.97	105.25	70.736	4.11	3.94	2.06	0.79	1.56	1.9	2.027	2.717	2.22	2.18	4.31	3.21	3.16	4.84	232.02	194.27	33.7	36.72	682.12	594.79
58	56	M	M	1	0	0	1	1	1	1	1	1.4	2.7	153	124	80	65	27.57	24	14	13	2235	1657	118.2	118.2	42.58	42.73	114.946	125.5	5.16	4.71	1.76	1.8	1.52	1.56	2.677	2.007	2.23	2.22	4.35	3.54	4.93	4.59	168.87	164.07	34.75	34.09	1236.84	1000.62
70	63	M	M	1	0	1	1	1	1	1	1	1.4	2.9	170	115	80	62	24.54	24.11	15	13	3900	1720	119.6	121.9	40.82	39.2	123.788	114.946	5.16	4.85	1.88	1.62	1.51	1.75	2.827	2.597	2.46	2.08	4.16	3.21	4.99	5.13	212.27	203.97	34.15	33.65	695.36	949.19
61	81	M	M	0	1	1	1	1	1	1	1	2.7	2.9	160	110	79	80	25.39	23.05	13	14	1890	1745	127.6	125.4	40.81	39.2	105.25	106.104	4.71	4.39	1.27	0.67	1.8	1.56	2.027	2.927	2.22	2.37	3.91	3.46	4.8	4.86	182.07	150.87	37.22	34.7	1044.44	566.28
61	47	M	F	0	0	1	1	1	0	1	0	1.7	2.8	156	130	78	69	22.43	25	14	13	4800	1919	116.3	124.6	39.52	40.96	114.946	97.262	5.02	4.39	1.05	1.62	1.87	1.85	1.787	2.007	2.21	2.22	4.24	3.21	4.91	3.09	221.97	203.97	33.7	33.92	1101.60	1126.37
39	81	F	M	0	0	1	1	0	1	0	1	1.8	2.5	145	128	79	66	26.95	26.4	11	12	4815	1746	125.4	125.6	41.58	42.36	123.788	70.736	4.37	4.99	0.93	1.5	1.52	1.56	1.787	2.597	2.22	2.36	3.91	3.26	4.93	5.19	168.87	150.87	37.21	33.2	1016.22	1104.71
42	54	M	F	0	0	0	1	1	0	1	0	1.4	2.4	138	111	79	62	25.74	24.51	13	14	4545	1931	124.6	131.2	42.21	39.75	125.5	125.5	4.11	4.39	2.06	1.8	1.52	1.57	2.027	2.977	2.46	2.46	4.12	3.42	4.66	5.13	182.07	194.27	34.75	34.25	1085.53	1070.03
48	65	M	M	0	0	1	1	1	1	1	1	1.7	2.1	150	120	95	80	25.78	23.72	11	12	6030	2000	121.9	137.8	42.62	41.73	141.472	61.894	4.88	3.82	1.88	1.24	1.51	1.92	2.757	2.247	2.18	2.34	3.91	3.26	5.2	3.09	182.47	164.47	34.15	33.65	1105.88	580.04
58	52	F	M	0	1	1	1	0	1	0	1	1.4	2.4	151	115	75	60	25.74	25.3	12	13	3900	1728	124.8	118.2	42.58	42.73	141.472	123.788	4.56	4.85	1.5	0.67	1.7	1.85	2.377	2.007	2.36	2.18	4.09	3.21	3.16	4.59	212.27	164.07	34.42	36.72	977.78	606.03
61	63	M	M	1	0	1	1	1	1	1	1	1.4	2.3	152	130	95	68	25.61	23.72	13	14	4500	1633	118.2	119.6	40.81	39.67	106.104	97.262	4.08	3.91	1.65	0.79	1.85	1.9	2.027	2.897	2.28	2.22	3.91	3.53	4.66	4.82	182.07	150.87	35.2	33.2	1005.35	1126.37
46	91	M	M	0	0	1	1	1	1	1	1	1.4	2.5	130	113	75	80	25.87	26.4	12	13	5250	1736	119.6	121.9	40.82	42.77	114.946	79.578	5.02	4.99	1.76	0.87	1.8	1.85	2.707	2.247	2.22	2.33	4.16	3.26	4.81	4.73	191.77	194.27	35.56	36.71	746.27	608.49
56	46	M	F	0	1	0	0	1	0	1	0	1.4	2.3	150	120	95	67	25.6	24.51	14	13	4635	2022	125.6	125.4	41.58	42.36	114.946	97.262	4.11	4.71	0.93	0.77	1.51	1.75	1.787	2.007	2.33	2.28	4.24	3.42	4.93	4.59	168.87	194.27	36.39	34.25	1355.26	1210.85
45	75	M	M	1	0	1	1	1	1	1	1	2.1	2.4	154	119	76	62	28.69	25.3	13	14	4920	2012	116.3	124.6	42.38	40.96	97.262	88.42	4.16	3.91	1.88	1.8	1.56	1.57	2.027	2.927	2.34	2.21	4.31	3.26	4.8	3.09	212.27	194.27	34.15	33.65	1059.52	907.16
41	58	M	M	0	0	1	1	1	1	1	1	1.8	3.0	140	129	77	64	21.88	25.78	12	13	4605	1821	125.4	125.6	42.21	40.97	114.946	125.5	5.16	4.99	2.06	1.5	2.01	1.9	2.377	2.247	2.22	2.22	4.24	3.21	3.16	5.13	182.07	194.27	35.2	33.92	695.36	756.14
51	63	F	M	1	0	0	1	0	1	0	1	1.4	2.7	138	112	78	70	22.3	22.3	15	13	3315	1633	124.6	132.6	41.58	39.53	141.472	106.104	3.99	3.94	1.05	0.79	1.87	1.57	2.677	2.007	2.46	2.21	4.12	3.46	4.66	5.14	221.97	164.07	37.25	34.7	547.06	826.44
72	60	M	M	1	1	1	1	1	1	1	1	1.4	1.7	133	120	76	72	28.75	26.02	15	13	5445	2000	132.6	118.2	39.05	39.75	150.314	114.946	4.88	4.54	1.05	1.24	1.51	1.85	2.027	2.597	2.46	2.36	3.91	3.65	4.8	4.84	227.27	214.02	34.59	35.06	1092.72	1297.01
66	63	M	M	1	0	1	1	1	1	1	1	1.6	2.7	136	121	77	73	27	26.09	14	13	4605	1913	121.9	119.6	42.58	42.73	125.5	61.894	4.11	4.85	1.03	0.79	1.7	1.75	1.787	2.247	2.18	2.22	4.09	3.21	5.14	5.07	212.27	194.27	35.2	35.89	1144.44	930.35
49	49	M	F	0	1	1	0	1	0	1	0	1.4	2.5	144	130	78	80	24.9	24.51	12	12	6135	1805	118.2	127.6	40.81	42.36	114.946	125.5	5.02	4.99	1.88	1.5	1.68	1.56	2.377	3.017	2.22	2.18	4.23	3.54	4.66	5.13	182.07	164.07	33.7	33.65	1165.56	965.41
46	81	M	M	0	0	1	1	1	1	1	1	1.4	2.4	128	122	79	70	27.57	24.39	14	12	4830	1914	119.6	116.3	42.62	42.53	106.104	88.42	5.16	3.82	0.93	0.79	1.8	2.06	1.787	2.007	2.08	2.46	4.31	3.21	4.93	3.09	236.72	150.87	37.21	34.7	611.84	608.49
47	61	M	M	0	1	1	0	1	1	1	1	1.7	2.1	150	130	80	60	28.86	26.09	14	12	3900	1900	116.3	125.4	42.21	42.36	88.42	106.104	4.71	4.54	2.06	1.62	1.51	1.56	2.377	2.247	2.37	2.37	4.24	3.61	4.91	4.59	212.27	203.97	35.56	36.75	682.12	682.18
45	47	M	F	0	0	1	1	1	0	1	0	1.4	2.5	152	112	81	80	29.54	25.6	13	14	5775	1940	121.6	124.6	40.82	41.73	125.5	70.736	4.08	4.52	1.65	1.39	1.75	1.92	1.787	2.977	2.36	2.22	4.35	3.21	4.66	4.86	168.87	173.77	35.2	34.09	724.36	960.25
59	76	M	M	0	1	1	1	1	1	1	1	2.6	2.9	138	124	82	66	30	26.83	14	12	4890	2000	125.4	121.9	42.21	42.53	97.262	79.578	4.56	3.94	1.5	1.62	1.87	1.92	2.027	2.007	2.33	2.34	3.91	3.26	4.93	4.59	221.97	150.87	33.7	34.7	1245.03	1207.57
47	81	M	M	0	0	1	1	1	1	1	1	1.4	2.2	133	115	78	80	28.4	24.51	14	12	4590	1904	124.6	124.8	39.52	42.77	150.314	125.5	5.16	4.39	1.88	0.67	2.01	1.85	1.787	2.247	2.22	2.08	4.12	3.54	4.8	5.14	182.07	150.87	34.15	33.2	1024.88	895.76
66	55	M	M	0	1	1	0	1	1	1	1	1.4	2.5	143	110	87	69	27.85	26.09	14	12	4590	1719	137.8	118.2	42.58	40.96	70.736	106.104	4.71	4.54	1.76	1.62	1.7	1.75	2.377	2.597	2.21	2.21	3.91	3.42	3.16	5.13	191.77	203.97	34.75	36.71	1245.03	1217.46
49	72	M	F	1	0	0	1	1	0	1	0	1.8	2.1	154	129	75	63	25.93	26.02	13	14	3690	1643	125.6	119.6	39.6	42.53	106.104	114.946	4.08	3.82	0.93	0.77	1.85	1.61	2.027	2.007	2.22	2.18	4.24	3.39	5.2	4.73	168.87	150.87	37.22	35.06	596.15	606.03
49	76	M	M	0	1	1	1	1	1	1	1	1.6	2.5	140	124	76	70	25.74	25.78	12	12	4650	2013	118.2	125.6	40.81	40.97	167.998	114.946	5.16	4.85	2.06	1.62	1.8	1.92	2.027	2.247	2.28	2.28	4.17	3.26	5.26	4.84	224.05	194.27	37.24	34.7	1245.03	605.75
35	65	M	M	0	0	1	1	1	1	1	1	1.8	2.0	153	115	78	72	27.67	24.39	14	12	5115	1656	119.6	116.3	42.38	42.36	97.262	167.998	5.16	3.91	1.05	1.01	1.68	1.9	2.757	2.897	2.18	2.22	3.91	3.21	4.66	4.92	182.07	150.87	36.59	33.2	583.33	636.89
59	63	M	M	0	1	1	1	1	1	1	1	2.1	2.7	130	110	75	74	23.63	26.02	14	12	4620	1824	116.3	125.4	41.58	42.53	125.5	88.42	4.71	4.54	1.88	1.62	1.85	1.75	1.787	2.007	2.46	2.46	4.16	3.46	4.99	4.86	182.47	150.87	34.15	33.65	1105.88	1052.79
54	53	M	M	0	0	1	0	1	1	1	1	2.5	2.4	154	127	79	80	23.05	26.09	15	13	5100	1813	121.9	124.6	42.38	41.69	106.104	114.946	4.11	3.94	1.5	0.77	1.51	1.85	2.027	2.977	2.34	2.36	4.24	3.26	4.66	4.59	212.27	194.27	34.42	34.25	891.89	973.29
79	81	F	F	1	1	0	1	0	0	0	0	1.7	2.6	138	125	95	69	32.92	26.17	13	14	3705	1730	125.4	118.2	40.82	42.73	123.788	79.578	5.16	4.99	1.65	1.62	1.68	2.06	2.797	2.007	2.37	2.33	4.31	3.47	3.16	4.52	221.97	173.77	35.2	36.72	1044.44	1054.68
47	73	M	M	0	0	1	0	1	1	1	1	1.4	2.7	132	115	76	66	26.34	23.05	12	13	2730	2000	124.6	119.6	42.38	39.75	106.104	70.736	5.02	3.99	0.93	0.79	1.7	1.56	1.787	2.597	2.36	2.37	3.91	3.26	5.21	3.09	232.02	164.07	34.59	36.74	977.78	1140.54
76	62	M	M	0	0	1	1	1	1	1	1	1.4	2.0	120	130	80	63	26	24	13	14	5820	1904	131.2	137.8	39.6	39.67	125.5	88.42	4.71	4.54	2.06	1.62	1.85	1.56	2.027	2.007	2.22	2.18	4.12	3.54	4.66	4.84	168.87	203.97	37.49	36.09	1005.35	968.81
89	76	F	F	0	1	0	1	0	0	0	0	1.4	2.6	125	124	81	80	22.13	25.3	13	14	6735	2127	132.6	116.3	42.38	42.77	132.63	97.262	4.16	4.2	1.88	1.8	1.51	1.92	2.757	2.717	2.33	2.22	4.35	3.21	4.8	5.13	212.27	206.05	34.15	34.7	1144.44	561.86
54	66	M	M	1	0	1	1	1	1	1	1	2.6	2.8	122	115	95	69	27.03	26.4	12	13	6000	4500	118.2	124.6	42.58	42.36	97.262	79.578	4.88	4.85	1.27	1.62	1.8	1.75	2.377	2.977	2.18	2.36	4.31	3.61	5.2	5.14	182.07	194.27	35.56	34.25	1119.05	1217.46
77	91	M	M	0	0	1	1	1	1	1	1	2.1	2.4	121	110	80	66	28.27	25.2	13	14	6330	5250	119.6	121.9	42.38	42.53	70.736	125.5	4.11	3.94	1.76	1.39	1.87	1.9	2.027	2.247	2.21	2.34	4.23	3.26	4.66	4.84	191.77	150.87	33.7	34.75	516.67	1332.85
69	61	M	M	0	1	1	0	1	1	1	1	2.4	2.4	120	129	78	63	26.95	26.09	13	14	5490	4635	116.3	125.4	42.62	41.73	97.262	70.736	4.08	4.99	1.5	1.62	1.52	1.85	1.787	2.927	2.23	2.23	4.24	3.46	4.93	4.59	212.27	214.02	34.42	33.92	935.32	1210.85
71	81	M	M	1	0	1	1	1	1	1	1	1.8	2.5	128	124	82	80	24.81	26.02	15	13	4905	4920	121.9	124.8	42.38	40.96	123.788	97.262	3.99	4.54	0.93	1.5	2.01	1.92	2.497	2.597	2.22	2.46	4.31	3.54	4.91	4.86	168.87	164.07	35.2	36.99	882.35	590.46
71	55	M	M	0	0	1	1	1	1	1	1	2.5	2.3	154	115	95	60	27.2	23.44	12	13	6300	4605	124.6	118.2	39.6	40.97	114.946	106.104	5.02	3.91	1.88	1.62	1.7	1.57	2.027	2.247	2.37	2.23	4.12	3.42	4.93	4.73	227.27	203.97	33.7	33.2	1178.81	989.81
44	89	F	F	0	0	0	1	0	0	0	0	1.9	2.3	122	110	94	80	26.34	23.6	13	14	5490	3315	125.4	132	42.38	42.73	79.578	79.578	4.56	4.39	2.06	1.24	1.85	1.75	2.677	3.047	2.28	2.18	3.91	3.21	3.16	3.09	212.27	164.07	37.49	34.7	1016.22	1217.46
51	84	M	M	1	1	1	1	1	1	1	1	1.4	2.0	152	130	93	63	28.27	24.11	13	14	5220	5445	125.6	129	40.81	42.36	150.314	125.5	4.11	4.99	1.05	1.8	1.8	1.92	1.787	2.597	2.22	2.22	4.16	3.26	4.66	4.59	182.07	203.17	37.24	33.92	695.36	910.58
59	75	M	M	0	1	1	1	1	1	1	1	1.7	2.3	130	112	75	69	27.5	23.2	12	13	5250	4605	121.6	112.8	42.38	42.36	132.63	114.946	5.16	3.82	1.65	1.52	1.56	1.85	2.027	2.247	2.46	2.21	4.24	3.53	5.2	4.74	221.97	194.27	33.7	33.2	1144.44	541.16
43	46	M	M	1	0	1	0	1	1	1	1	2.1	2.4	153	124	76	80	25.69	24.39	12	13	4590	6135	118.2	117.5	42.38	42.53	125.5	106.104	4.37	4.85	1.27	0.79	1.68	1.73	2.377	2.977	2.18	2.28	4.12	3.39	4.89	4.92	224.05	150.87	36.39	34.25	1245.03	1332.85
42	81	M	M	0	0	1	1	1	1	1	1	2.6	2.5	134	115	80	69	27.24	24.54	13	14	4815	4830	119.6	125.4	41.54	39.75	97.262	70.736	5.16	4.71	1.88	0.67	1.85	1.92	2.757	2.597	2.22	2.18	4.19	3.54	3.16	3.09	212.27	164.07	34.15	34.75	497.33	1036.14
57	68	M	M	1	0	1	1	1	1	1	1	1.9	3.0	132	110	79	66	29.22	22.36	15	13	4800	3900	137.8	122.3	39.6	39.2	114.946	88.42	4.88	4.99	0.93	1.39	1.7	1.56	2.027	2.247	2.36	2.36	4.24	3.26	4.66	5.14	168.87	164.07	33.7	33.2	1092.72	1054.68
59	81	M	M	1	0	1	0	1	1	1	1	2.1	2.8	135	116	78	63	29.2	22.53	12	13	5550	5775	116.3	121.9	40.82	42.36	110.525	106.104	4.71	3.99	2.06	1.8	1.51	1.56	1.787	2.007	2.34	2.37	3.91	3.21	4.8	4.59	221.97	150.87	34.42	33.92	1074.29	724.95
43	61	M	M	0	1	1	1	1	1	1	1	2.4	2.4	136	130	94	80	29.11	25.6	13	13	3030	4890	124.6	116.3	42.58	42.73	119.367	61.894	3.97	4.85	1.76	1.5	1.87	1.75	2.427	2.977	2.22	2.22	4.24	3.46	5.14	4.86	182.07	203.97	35.2	34.25	1165.56	590.46
54	67	M	M	0	0	1	1	1	1	1	1	2.5	2.3	125	112	75	69	26	26.09	12	13	4800	4590	121.9	125.6	39.05	40.97	111.4092	88.42	5.16	3.91	1.78	0.67	1.8	1.57	2.027	2.247	2.21	2.46	4.12	3.42	4.93	4.84	212.27	194.27	33.7	33.2	550.80	541.16
47	81	M	M	0	0	1	1	1	1	1	1	1.4	2.8	151	124	95	66	26.48	25.6	12	13	5160	4590	125.4	137.8	39.05	42.36	106.104	125.5	4.32	3.94	1.88	0.77	1.85	1.56	2.377	2.007	2.36	2.34	4.26	3.54	5.2	3.09	236.72	206.05	34.15	36.99	1245.03	1219.46
41	66	M	M	0	0	1	1	1	1	1	1	1.7	2.7	152	115	80	63	28.86	23.72	13	13	3960	1703	124.8	127.6	40.81	39.75	106.104	97.262	4.11	4.54	2.06	0.87	1.51	1.85	1.787	2.897	2.46	2.36	4.12	3.26	4.66	4.73	221.97	164.07	34.59	33.92	1144.44	1054.68
47	74	F	F	1	0	0	1	0	0	0	0	2.4	2.3	125	110	95	80	26.95	22.92	13	13	4560	1857	118.2	118.2	42.21	39.2	123.788	44.21	4.71	4.85	0.93	1.62	1.52	1.92	2.027	2.247	2.18	2.33	3.91	3.26	4.96	5.07	182.07	214.02	33.7	33.2	547.06	780.94
79	48	M	M	0	0	1	0	1	1	1	1	2.0	2.5	154	128	94	69	24.81	24.8	13	13	4605	2000	119.6	125.4	39.6	39.67	125.5	132.63	5.02	3.82	1.03	1.8	2.01	1.56	2.677	2.007	2.22	2.22	4.09	3.21	4.66	4.59	168.87	150.87	36.39	34.25	1236.84	973.29
51	58	F	F	0	1	0	1	1	0	0	0	2.6	2.2	129	124	81	66	20.82	22.13	13	13			125.6	124.6	41.58	42.53	125.4	132.6	4.71	4.85	1.05	1.62	1.7	1.8	2.377	3.047	2.21	2.18	4.24	3.49	4.91	5.14	191.77	194.27	35.56	34.09	604.28	1140.54
61	71	M	M	1	0	1	1	1	1	1	1	2.4	2.5	130	115	83	63	25	26.83	12	13			124.6	124.8	42.58	41.73	119.6	125.4	5.02	4.99	1.88	0.67	1.51	1.75	2.027	2.247	2.33	2.21	4.31	3.61	4.8	3.09	212.27	164.07	33.7	33.2	617.65	531.63
73	65	F	M	0	0	1	1	1	1	0	1	1.4	1.3	134	110	77	70	24.91	26.17	13	13			116.3	121.9	39.52	42.53	132.6	118.2	4.71	3.94	2.06	1.62	1.56	1.56	1.787	2.007	2.22	2.08	4.12	3.21	4.93	4.86	224.05	203.97	34.42	33.92	1074.29	1217.46
64	60	M	F	0	0	1	1	1	1	1	0	1.6	3.0	139	130	79	80	27.6	23.94	11	12			118.2	125.6	36.59	36.99	124.6	116.3	5.16	4.54	1.65	0.79	1.8	1.57	2.757	2.597	2.18	2.46	3.91	3.54	4.99	4.59	232.02	150.87	35.2	34.25	941.18	1151.97
64	52	F	F	1	1	1	0	1	1	0	0	2.5	2.7	140	124	76	69	27.92	22.4	13	13			132.6	122.3	35.2	35.06	131.2	137.8	4.71	4.99	0.93	1.62	2.01	1.56	1.787	2.247	2.37	2.37	4.19	3.26	4.66	4.73	182.07	173.77	33.7	33.2	1101.60	685.41
42	58	M	F	0	0	0	1	0	0	1	0	1.6	1.7	115	115	80	66	28.86	22.53	13	13			125.4	118.2	34.15	33.2	125.6	125.6	4.11	3.99	1.76	1.39	1.51	1.85	2.677	2.007	2.22	2.22	4.24	3.42	5.2	4.82	212.27	164.07	37.24	35.06	1245.03	590.46
45	70	M	F	0	0	1	0	1	1	1	0	1.7	2.7	110	110	75	63	28.46	26.02	11	12			118.2	127.6	37.24	34.7	137.8	119.6	4.71	4.71	1.88	1.62	1.52	1.92	2.377	2.977	2.08	2.28	4.16	3.26	4.91	4.73	221.97	150.87	34.15	33.92	611.84	672.59
51	64	F	M	1	0	1	1	1	1	0	1	1.9	2.5	154	130	76	80	25.37	24.54	13	14			121.9	125.4	34.42	36.99	119.6	132.6	4.88	4.85	2.06	1.5	1.87	1.9	2.027	2.247	2.22	2.18	4.35	3.65	4.93	3.09	213.94	194.27	33.7	33.2	993.38	1079.00
61	47	F	F	0	1	0	1	0	0	0	0	1.8	2.4	116	125	77	69	27.92	20.68	11	12			118.2	119.6	37.49	36.09	125.4	124.8	4.71	3.94	1.76	1.62	1.7	1.57	1.787	2.007	2.28	2.36	3.91	3.21	3.16	4.84	168.87	173.77	37.21	34.25	1044.44	608.49
64	47	F	F	1	0	1	1	1	1	0	0	2.4	2.1	153	115	78	66	29.54	23.17	13	14			119.6	132.6	35.2	34.7	122.3	116.3	5.02	4.39	1.03	1.8	1.68	1.75	2.707	2.597	2.46	2.34	4.31	3.54	4.66	3.09	191.77	164.07	35.56	34.7	841.06	1116.50
49	55	F	F	1	1	1	1	1	1	0	0	2.4	2.5	126	129	75	63	24.9	25.6	11	12			121.6	124.6	34.15	33.65	127.6	118.2	4.71	3.91	0.93	1.62	1.51	1.85	2.757	2.247	2.22	2.21	4.12	3.26	4.66	5.19	168.87	203.97	33.7	33.65	617.65	1070.03
59	75	M	F	1	0	1	0	1	1	1	0	2.1	2.9	152	112	76	70	24.63	25.19	11	12			116.3	131.2	34.75	36.74	118.2	132.6	4.11	4.54	1.88	0.67	1.8	1.92	1.787	2.007	2.36	2.22	4.23	3.21	4.89	4.59	212.27	194.27	34.15	33.2	1245.03	849.27
48	55	F	F	0	1	1	1	1	1	0	0	1.6	2.2	140	124	79	80	25.65	24.9	13	14			124.6	125.6	34.59	33.92	131.2	125.4	4.71	4.39	2.06	1.62	1.52	2.06	2.377	2.927	2.18	2.37	4.31	3.42	4.8	4.86	182.07	209.27	34.42	34.25	1101.60	1104.71
47	63	F	M	0	1	1	1	1	1	0	1	2.4	2.5	139	115	75	69	26.17	22.4	13	14			137.8	137.8	34.42	36.99	124.6	116.3	5.16	4.99	1.76	1.24	1.85	1.57	2.027	2.247	2.22	2.46	3.91	3.26	5.2	4.84	212.27	195.94	33.7	33.65	1165.56	1134.41
56	48	M	F	1	1	0	1	0	0	1	0	1.8	2.1	135	110	76	66	26.25	23.68	13	14			125.4	119.6	35.56	34.7	121.6	113.6	4.71	3.94	1.05	1.52	2.01	1.73	2.677	2.007	2.34	2.18	4.24	3.21	3.16	4.59	221.97	164.07	34.75	36.09	1016.22	527.96
75	54	F	F	0	1	1	1	1	1	0	0	1.7	2.5	132	125	79	63	28.27	25.3	11	12			118.2	125.4	34.15	33.65	125.6	125.6	4.88	3.82	2.06	1.62	1.7	1.56	1.787	2.977	2.23	2.33	4.17	3.39	4.93	4.73	212.27	150.87	35.2	33.2	1059.52	1207.57
55	47	F	F	1	0	1	1	1	1	0	0	1.4	2.0	130	115	80	80	27.6	22.4	11	12			125.6	122.3	33.7	34.25	125.4	127.6	4.11	4.85	1.88	0.79	1.8	1.75	2.497	2.247	2.22	2.23	4.26	3.21	5.14	4.74	182.47	194.27	33.7	33.92	572.22	617.89
64	59	M	M	0	0	1	0	1	1	1	1	2.6	2.7	125	128	95	69	27.24	22.53	13	14			121.9	127.6	35.2	34.09	118.2	132.6	5.02	4.71	0.93	1.8	1.51	1.56	1.787	2.597	2.18	2.22	4.12	3.46	4.93	3.09	224.05	203.97	34.59	33.65	1245.03	1022.75
49	67	F	F	1	0	1	1	1	1	0	0	2.1	2.4	129	125	80	66	26.95	23.42	11	12			122.3	118.2	34.59	33.92	132.6	118.2	4.71	4.99	1.65	1.62	1.52	1.85	2.377	2.897	2.36	2.36	3.91	3.21	4.66	4.84	212.27	164.07	36.59	34.25	679.14	824.83
55	54	F	F	0	0	1	1	1	1	0	0	1.8	2.6	122	115	95	63	25.87	25.3	14	13			124.6	131.2	37.49	35.06	137.8	121.9	5.16	3.94	1.78	0.67	1.7	1.56	2.027	2.247	2.37	2.23	4.24	3.26	5.2	5.07	227.27	206.05	34.42	33.2	1364.24	1070.03
47	60	M	F	0	1	1	0	1	1	1	0	2.4	2.7	115	130	95	80	25.74	24.54	14	13			116.3	124.6	34.15	33.65	127.6	125.4	4.11	4.99	1.76	1.39	1.51	1.57	2.757	2.007	2.46	2.28	4.12	3.54	5.21	4.86	168.87	150.87	35.56	34.7	1236.84	605.75
60	52	F	M	1	0	1	1	1	1	0	1	1.6	2.0	110	117	79	69	27.08	22.92	14	13			132.6	121.6	34.42	33.2	131.2	124.6	4.88	4.71	1.88	1.62	1.51	1.56	1.787	2.717	2.22	2.46	4.16	3.26	4.66	3.09	182.07	194.27	34.15	33.65	695.36	1332.85
68	47	M	F	0	1	1	1	1	1	1	0	1.7	2.6	119	130	80	66	24.61	24	14	13			121.9	125.6	35.2	34.7	124.6	125.6	4.71	4.39	1.05	1.5	1.52	1.61	2.757	2.247	2.08	2.18	4.31	3.21	3.16	4.59	191.77	164.47	37.25	34.25	970.59	1054.68
55	58	M	M	1	0	1	1	1	1	1	1	2.5	2.8	120	112	79	63	26.8	22.13	14	12			125.4	125.6	36.59	34.09	116.3	131.2	5.02	4.85	0.93	0.77	1.87	1.56	1.787	2.977	2.33	2.22	3.91	3.61	4.93	4.73	212.27	164.07	37.21	33.2	1245.03	826.44
61	57	M	F	0	1	1	0	0	1	1	0	2.4	2.4	125	124	80	80	27.27	22.06	13	14			118.2	113.6	37.24	36.99	121.6	137.8	4.11	4.54	1.03	1.62	1.8	2.06	2.377	2.597	2.22	2.34	4.24	3.21	3.16	4.92	212.27	203.97	36.39	34.09	977.78	1036.14
52	62	F	M	1	0	0	1	1	1	0	1	2.6	2.4	121	115	81	66	26.17	25	13	14			125.6	125.4	34.15	33.65	125.6	118.2	4.08	4.99	1.65	1.8	1.51	1.56	2.027	2.247	2.18	2.33	4.09	3.42	4.8	4.59	221.97	194.27	34.42	33.65	1364.24	973.87
48	62	F	M	0	1	1	1	1	1	0	1	2.4	2.5	126	110	76	69	29.11	21.37	11	12			131.2	119.6	35.2	33.92	122.3	119.6	4.71	4.99	1.88	0.67	1.85	1.61	2.677	3.017	2.23	2.22	4.12	3.49	4.66	4.89	212.27	150.87	34.15	33.92	1005.35	527.96
58	67	F	M	1	0	1	1	1	1	0	1	1.8	2.3	130	119	79	80	25.87	24.4	14	12			124.6	121.6	34.42	34.7	119.6	121.9	4.56	3.94	1.88	1.62	1.8	1.56	2.377	2.007	2.34	2.28	4.35	3.54	5.2	3.09	168.87	164.07	34.59	33.2	993.38	1217.46
57	66	F	F	0	0	1	1	1	0	0	0	1.6	2.3	138	129	80	70	28.75	24.39	11	12			121.6	113.6	37.49	36.09	121.9	125.4	5.02	4.71	1.05	0.87	1.51	1.85	2.027	2.247	2.22	2.08	3.91	3.26	4.91	5.14	212.27	173.77	37.21	34.25	604.28	1126.37
63	63	F	F	1	1	1	1	0	1	0	0	1.9	2.0	154	124	95	60	26.42	26.09	13	14			132.6	125.4	33.7	36.74	127.6	123.788	4.11	3.94	1.65	0.79	1.7	1.75	1.787	2.597	2.36	2.36	4.23	3.53	4.81	4.73	182.07	209.27	37.22	34.7	1245.03	685.41
63	77	F	F	0	0	0	1	1	0	0	0	1.7	2.3	125	115	79	80	25.74	22.53	14	12			137.8	118.2	34.15	33.65	119.6	123.788	4.71	4.85	2.06	1.62	1.8	1.9	2.027	2.977	2.46	2.18	4.31	3.21	4.93	4.86	212.27	164.07	37.21	34.25	522.39	930.35
67	60	F	F	0	1	1	1	1	1	0	0	1.6	2.4	130	110	78	63	26.48	27.24	11	12			125.4	125.4	37.22	34.7	131.2	141.472	4.56	4.54	1.88	1.05	1.51	1.85	1.787	2.247	2.28	2.46	4.12	3.26	3.16	4.73	212.27	194.27	33.7	33.2	1144.44	826.44
67	70	F	M	1	1	1	1	0	1	0	1	2.4	2.5	122	128	76	68	26.95	26.09	14	12			125.6	116.3	34.59	33.92	118.2	141.472	4.88	3.94	1.92	0.93	1.67	2.02	1.82	2.11	2.22	2.22	4.19	3.61	4.66	4.59	168.87	173.77	36.59	35.06	1236.84	1022.75
64	72	M	F	0	1	1	1	1	1	1	0	2.5	3.0	153	124	75	80	27.41	24.8	14	12			121.9	119.6	33.7	36.99	124.6	106.104	4.71	4.71	1.88	0.67	1.52	1.75	1.787	2.007	2.18	2.37	3.91	3.42	5.2	4.92	221.17	203.97	35.56	33.92	615.89	608.49
78	53	F	F	0	0	1	1	1	1	0	0	2.4	2.8	115	115	80	69	30	24.61	13	14			124.6	114.9	37.21	33.2	125.6	114.946	5.16	4.54	1.03	0.77	1.87	1.61	2.027	2.597	2.37	2.33	4.09	3.54	5.14	4.84	212.27	164.07	34.59	34.25	1035.29	1325.50
61	55	F	M	0	1	0	0	1	0	0	1	2.1	2.4	119	110	81	66	29.96	22.43	14	12			131.2	116.3	34.15	33.65	132.6	114.946	4.16	4.85	1.88	1.62	1.8	1.57	2.757	2.247	2.22	2.34	4.12	3.21	4.66	3.09	182.07	150.87	37.21	33.2	1005.35	1140.54
70	54	M	F	1	0	1	1	1	1	1	0	1.4	2.3	110	129	80	63	25.39	25.78	11	12			122.3	125.4	34.42	36.72	121.9	97.262	4.88	4.54	1.5	1.01	1.85	1.56	2.707	2.717	2.21	2.28	4.24	3.39	4.8	5.07	227.27	206.05	34.15	36.74	916.67	1047.57
72	60	F	F	0	1	1	1	1	1	0	0	2.2	2.8	154	117	75	80	24.91	22.8	14	12			116.3	125.6	33.7	34.09	118.2	114.946	5.02	3.94	1.88	1.8	1.8	1.85	2.027	2.977	2.18	2.18	4.31	3.21	4.99	4.92	221.97	194.27	37.49	33.65	682.12	1217.46
53	56	M	F	0	1	0	1	0	1	1	0	1.8	2.7	152	130	95	70	29.58	22.13	11	12			125.6	127.6	34.75	33.2	137.8	141.472	4.11	4.54	1.76	1.62	1.7	1.92	1.787	2.247	2.22	2.22	3.91	3.47	3.16	3.09	232.02	150.87	34.75	34.7	1016.22	1151.97
56	58	M	M	0	1	1	1	1	1	1	1	1.7	2.3	151	125	75	60	30	25.6	14	12			125.4	125.4	37.24	36.71	137.8	150.314	4.56	4.99	1.88	0.79	1.52	1.57	2.677	2.597	2.08	2.21	4.23	3.54	4.91	4.59	212.27	214.02	35.2	35.89	1355.26	608.49
55	66	M	F	1	0	1	1	1	1	1	0	2.6	1.2	139	115	79	80	26.09	25.78	13	14			118.2	116.3	34.15	33.65	125.6	97.262	4.08	4.54	2.06	1.39	1.85	1.57	2.027	2.897	2.37	2.46	4.12	3.42	5.2	4.86	182.07	164.07	36.39	33.92	679.14	580.04
61	53	F	M	0	0	1	0	1	0	0	1	2.4	1.2	134	130	80	63	25.74	24.39	13	14			121.9	119.6	34.75	33.92	124.6	114.946	4.71	4.71	1.65	1.62	1.52	1.56	2.377	2.247	2.22	2.36	4.16	3.21	4.66	4.84	212.27	194.27	34.15	33.65	1245.03	685.41
56	71	F	F	1	0	1	1	1	1	0	0	1.9	1.3	132	125	76	69	25	23.28	12	12			124.6	132.6	35.56	33.2	127.6	106.104	4.56	3.94	0.93	1.5	1.8	1.75	1.787	2.007	2.36	2.23	4.12	3.26	4.93	4.82	191.77	203.97	37.21	34.09	547.06	1022.75
58	52	F	M	0	1	1	0	1	1	0	1	1.6	1.3	130	115	78	80	23.75	24.8	13	14			132.6	125.4	34.42	34.25	121.9	88.42	5.16	4.85	1.65	1.8	1.7	1.9	2.027	2.597	2.46	2.37	3.91	3.21	5.2	4.73	168.87	218.72	33.7	34.7	938.09	1332.85
66	59	F	F	0	0	1	1	1	0	0	0	1.8	1.2	139	116	77	80	21.93	26.09	13	14			121.6	118.2	36.59	36.74	122.3	88.42	4.11	4.54	1.05	1.62	1.51	1.85	2.757	2.247	2.34	2.22	4.31	3.46	4.8	4.59	212.27	164.07	34.42	36.71	1236.84	853.76
53	49	F	M	1	1	1	1	1	1	0	1	2.4	1.1	154	130	75	69	32.07	26.02	13	14			118.2	116.3	34.15	33.65	124.6	97.262	3.99	4.99	1.03	1.24	2.01	1.56	2.497	3.017	2.18	2.18	4.35	3.54	5.21	3.09	212.27	150.87	37.49	33.65	993.38	541.16
71	82	F	F	0	0	1	1	1	1	0	0	2.6	1.3	125	124	75	66	22.13	27.24	12	12			116.3	137.8	37.22	34.25	116.3	150.314	5.02	3.94	1.65	0.87	1.51	1.61	1.787	2.977	2.22	2.08	3.91	3.65	4.66	4.59	182.07	194.27	34.59	35.06	1320.51	1134.41
53	66	F	M	1	0	1	1	1	1	0	1	1.7	1.3	153	115	79	63	29.69	25.78	13	14			125.4	125.6	34.59	35.06	121.6	70.736	4.88	4.71	2.06	1.62	1.87	2.06	2.377	2.247	2.33	2.34	4.31	3.61	3.16	5.07	227.27	164.47	37.24	33.92	1044.44	1217.46
60	66	M	F	0	0	1	1	1	1	1	0	2.1	1.3	130	110	75	80	27.85	23.44	12	12			125.6	119.6	36.39	33.92	118.2	106.104	5.16	4.54	1.27	0.79	1.87	1.92	2.707	2.597	2.28	2.46	4.12	3.54	4.91	5.19	212.27	150.87	37.25	34.7	562.19	1207.57
50	58	F	M	0	0	0	0	0	0	0	1	1.9	1.2	153	129	76	67	27.57	23.58	12	12			127.6	132.6	34.42	36.09	119.6	167.998	4.11	4.85	1.03	1.8	1.8	1.56	1.787	2.927	2.21	2.21	4.24	3.42	4.66	3.09	182.07	164.07	34.42	33.65	1092.72	960.25
83	71	F	F	1	0	1	1	1	1	0	0	2.2	1.2	152	124	80	65	29.11	23.32	13	14			121.9	124.8	33.7	33.65	125.6	97.262	5.16	3.94	1.65	1.62	1.7	1.75	2.377	2.247	2.22	2.22	4.17	3.21	5.2	4.86	221.97	203.97	36.59	36.09	547.06	595.22
66	64	F	M	0	1	0	1	0	1	0	1	1.6	1.3	115	115	94	80	26.8	22.13	13	14			132.6	116.3	37.21	36.72	124.6	123.788	4.88	3.91	2.06	0.67	1.56	1.73	2.757	2.007	2.21	2.36	3.91	3.49	4.93	4.73	168.87	194.27	35.56	33.2	705.56	608.49
67	60	F	F	1	1	1	0	1	1	0	0	1.4	1.2	120	110	75	68	27.34	23.58	13	14			118.2	118.2	35.2	34.09	118.2	106.104	4.56	4.54	1.05	1.5	1.87	1.85	2.027	3.017	2.36	2.18	4.12	3.53	3.16	4.84	221.97	203.17	37.24	34.09	1320.51	1031.63
65	59	M	M	1	0	0	1	1	0	1	1	2.5	1.3	132	115	80	67	26	24.22	13	14			124.6	132.6	35.56	35.89	132.6	123.788	5.02	4.39	2.06	1.62	1.85	1.56	1.787	2.247	2.22	2.28	4.31	3.39	5.26	3.09	168.87	206.05	34.59	33.65	1044.44	1325.50
